# Physical Activity Promotion in Malaysia: Challenges and Opportunities

**DOI:** 10.3389/fpubh.2020.536239

**Published:** 2020-10-27

**Authors:** Selina Khoo, Bee Koon Poh, Saiful Adli Suhaimi, Kar Hau Chong, Andrea Ramirez Varela

**Affiliations:** ^1^Centre for Sport and Exercise Sciences, University of Malaya, Kuala Lumpur, Malaysia; ^2^Centre for Community Health Studies (ReaCH), Faculty of Health Sciences, Universiti Kebangsaan Malaysia, Kuala Lumpur, Malaysia; ^3^Early Start, Faculty of Arts, Social Sciences and Humanities, University of Wollongong, Wollongong, NSW, Australia; ^4^College of Medicine, Universidad de los Andes, Bogota, Colombia

**Keywords:** physical activity, epidemiology, surveillance, policy, research

## Abstract

About three quarters of the Malaysian adult population are physically active. There has been growth in physical activity and health research since 2010, with most studies being observational in design and few included objective measures of physical activity. The Malaysian Ministry of Health has published physical activity guidelines, strategies and action plans aimed at promoting physical activity. Physical activity promotion activities have included national campaigns and programmes which target different populations. Further work that incorporates the WHO Global Action Plans on Physical Activity (GAPPA), as well as a more systemic approach is needed, to promote physical activity and a healthy lifestyle. High-level multi-stakeholder collaboration is required for continuing expansion and strengthening of research capacity, and for bridging the physical activity policy gaps in Malaysia.

## Introduction

According to the World Health Organization, non-communicable diseases (NCDs) are a major health concern, and accounted for 68% of global deaths in 2012 (1). Of these, 42% were premature deaths (under 70 years) with more than 80% occurring in low- and middle-income countries. NCDs are a major cause of death in Malaysia, an upper-middle income country in Southeast Asia having an estimated population of 32.6 million (2). Rapid socio-economic development and urbanization has influenced the Malaysian lifestyle and contributed to the rise of NCDs (3). Available data from the Ministry of Health Annual Reports shows that diseases associated with the circulatory system were the principal causes of death from 2010 to 2018, accounting for between 22 and 26% of deaths (4–12). Cancers were also among the five main causes of death at this time, accounting for between 11 and 14% of deaths. Population health surveys have shown an increasing trend in the prevalence of NCDs (e.g., diabetes mellitus and hypercholesterolemia) and NCD risk factors (13, 14). At present, the latest data reveals ~1.7 million Malaysians living with three major risk factors of NCDs (diabetes, hypertension, and high cholesterol) (15).

Physical activity has been shown to ameliorate the risk of NCDs (16–19). Despite the well-established benefits of physical activity, it is estimated that about one-third (31.1%) of the world's population is physically inactive (20). Physical inactivity is the fourth leading risk factor for global mortality and is estimated to cause 6% of deaths worldwide (21), with 5.3 million people dying every year as a result (22). In Malaysia, the rate of physical inactivity and the deaths attributed to it is greater than the global average. The latest population health survey reports that 25.1% of Malaysians are physically inactive (15) and physical inactivity is responsible for 16.4% of the country's deaths (23).

In 2012, in response to sweeping global physical inactivity, the Global Observatory for Physical Activity (24) (http://www.globalphysicalactivityobservatory.com/) was created. At the time, information on the overall picture of how well countries across the world were progressing on promoting physical activity was limited. In particular, little standardized information was available on surveillance or policy and research on physical activity. Consequently, GoPA! country profiles were created using a standardized methodology (25) (see Global Observatory for Physical Activity GoPA! website: http://www.globalphysicalactivityobservatory.com/country-cards/) with Malaysia included as a member.

In addition, a conceptual model (26) of country-level capacity for physical activity promotion was created which included information on periodic surveillance, physical activity policy implementation, and research productivity as its three main pillars.

This article aims to describe country-level capacity for physical activity promotion in Malaysia in the context of the current state of physical activity surveillance, policy, and research; and to discuss future steps corresponding to the WHO Global Action Plan on Physical Activity (GAPPA) and the United Nations Sustainable Development Goals.

## Malaysia's Capacity for Physical Activity Promotion: Description of Main Pillars and Implications

### Physical Activity Surveillance

Surveillance of physical activity in Malaysia is conducted by the Ministry of Health through National Health and Morbidity Surveys. First conducted in 1986, initial surveys were conducted every 10 years. However, from 2011 onwards, they were conducted annually differing in focus each year. Physical activity was incorporated in the surveys in 2011, 2015, and 2019. The first inclusion of a survey on physical activity was in 2006 using the short version of the International Physical Activity Questionnaire. The latest National Health and Morbidity Survey (NHMS 2019) reported that 74.9% of Malaysian adults were physically active, a figure higher than the prevalence reported in the previous three surveys (NHMS 2006: 56.3%; NHMS 2011: 64.8%; NHMS 2015: 66.5%) (13–15, 27). Males (77.9%) were found to be more physically active than females (71.8%).

### Physical Activity Plans and Policies

In 2016, the Ministry of Health developed national guidelines on physical activity in order to provide essential resources and guidance on physical activity and to achieve optimum health benefits. In 2017, the National Physical Activity Guidelines were published (28). Before this, physical activity recommendations were included as part of the general Malaysian Dietary Guidelines (29), and mirrored in the Malaysian Dietary Guidelines for Children and Adolescents (30). In 2016, the Ministry of Health developed the National Strategic Plan for Non-communicable Diseases to more effectively tackle an increasing prevalence of NCDs and NCD risk factors (3). Physical activity is specifically highlighted in this strategic plan.

Along with National Physical Activity Guidelines, in 2018, the Ministry of Health published the National Strategic Plan for Active Living, outlining strategies to encourage Malaysians to be more active (31). The Strategic Plan utilized a system-based approach to increase physical activity. Strategies suggested in the Strategic Plan are evidence-based and outline how to implement and monitor physical activity across four settings, namely, education, community, workplace, and healthcare. Three of the authors here (SK, BKP, SAS) were involved in various stages of the plan. Along with the National for Active Living, the Malaysian Health Promotion Board, under the auspices of the Ministry of Health, also promotes physical activity through the provision of grants to non-governmental organizations. Registered non-governmental organizations may apply for grants to conduct healthy lifestyle programs. During 2017, nearly half of the grants were allocated to physical activity programs (Mohd. Shukrimi Samsudin, personal communication, July 3rd, 2018).

The Ministry of Health has implemented various campaigns and programmes to encourage physical activity among the population (32). There had been national campaigns for cycling (in 2005) and 10,000 steps (in 2009). Physical activity has been a component of programmes at schools (Young Doctor), universities (Healthy University Students,) communities (Healthy Communities Empower the Nation), and the workplace (Active Citizens are Productive Citizens). Community Health Promotion Centres, established throughout the country, provide weekly exercise sessions and exercise consultation to the public.

### Physical Activity Research in Malaysia

Because Malaysia is part of the Global Observatory for Physical Activity GoPA! (33), it conducts regular physical activity research productivity updates. Collaborative research at two Malaysian universities discovered that the amount of physical activity research publications on the topic of physical activity and health per year in Malaysia had increased from just one in 1980 to 32 in 2019. The number of publications increased most rapidly in 2010, and the highest number of publications (n = 41) was recorded in 2016. In terms of study design, 212 (69.9%) of the studies were observational and 67 (22.1%) were experimental. The remaining 24 studies were qualitative (*n* = 16; 5.2%) or reviews of literature in Malaysia (*n* = 8; 2.6%). In the case of observational studies, 188 (62%) were cross-sectional, eight were (2.6%) longitudinal, and 16 (5.2%) were case-control studies.

**Figure 1:**
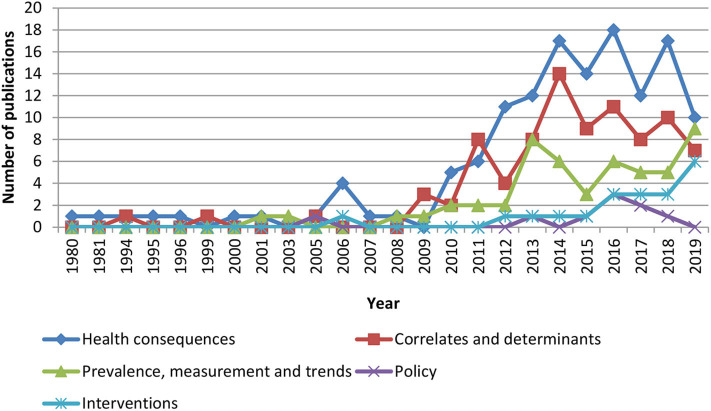
Types of physical activity research publications in Malaysia from 1980 to 2019.

As for study types (Figure 1), 52 (17.1%) were about prevalence, measurement and trends; 87 (28.7%) were about correlates and determinants; 36 (44.8%) were about health consequences; 20 (6.6%) were intervention studies, and eight (2.6%) were about policy. Target populations were diverse, with 72 studies (23.7%) conducted in children and adolescents (aged < 18 years); 130 studies (42.9%) in adults (aged ≥ 18 years and < 60 years), and 32 studies (10.5%) in older adults (aged ≥ 60 years). Sixty-five studies (21.4%) included two or more of these age categories. Only two studies were related to pregnant women (0.6%) and four studies (1.3%) did not specify the population studied. Sixteen studies (5.2%) were multi-national studies; and only 24 studies (7.9%) included objective measures of physical activity.

## Actionable Recommendations

A comprehensive overview of the results of current Malaysian capacity for physical activity promotion suggests the following recommendations:

1. To achieve the WHO universal goal of a 15% increase in prevalence and participation in physical activity by 2030 (34), the Ministry of Health has already developed the National Strategic Plan for Active Living which is currently being implemented by various Malaysian government agencies. The following strategies were highlighted in the Strategic Plan: an introduction of active transportation policies; the provision of safe and equitable access to recreation and physical activity; the provision of physical activity assessment and advice as a part of general healthcare services; ensuring regular physical activity in kindergartens and schools; working alongside communities in the provision of local physical activity programs, and the promotion of “sport for all” (31). These recommendations are purposively aligned with the WHO GAPPA 2018–2030, which aims to have one hundred million people being increasingly active by 2030 (34). GAPPA is based on four principles. These comprise: life course approach; equity; empowerment of peoples, families and communities, and, a human rights-based approach. Malaysia's effort in developing its own national plan of action for physical activity should encompass WHO GAPPA principles.
2. Physical activity research in Malaysia is developing. But, despite the number of publications increasing over the past decade, physical activity studies were mostly limited to observational studies, with nearly all being cross-sectional. Further research using objective measures is needed to provide an extensive picture of physical activity behavior, while additional research focusing on determinants of effective intervention programs would be beneficial in increasing physical activity among the population.
3. To address future research needs, it is critical to continue developing and strengthening Malaysian physical activity research capacity. Incorporation of physical activity, exercise, and sports studies in engineering, architecture, urban development, and town planning curricula ought to encourage greater inter-disciplinary effort in increasing physical activity amongst the population. Training postgraduate students and those in research institutions and universities in aspects of physical activity should improve the quantity and quality of research in the long term. Malaysian collaboration with countries more advanced in physical activity research will likely contribute to building necessary networking and research capacity.
4. Recent data confirming the lack of physical activity and the concomitant rise of NCDs in Malaysia has created a sense of urgency for all stakeholders to work together in ameliorating the crisis. Current programs that promote physical activity are done on an *ad-hoc* basis. It also appears that there is an absence of policy or well-planned strategy consistent with promoting the use of current physical activity guidelines intended to increase physical activity among Malaysians. Having a national physical activity action plan concordant with the WHO GAPPA will pave the way for a more physically active population in the near future. Ensuring collaboration between various government and non-governmental agencies (including urban planning and transportation, medical, health, and fitness) will result in smoother execution of a comprehensive physical activity plan.
5. It is incumbent on the Malaysian government to increase its efforts and to continue to fund physical activity programs and research. Development of a national plan of action for physical activity by the Ministry of Health should be prioritized and accelerated, as it is a policy precursor to supporting improvement in the indicators for physical activity. Moreover, Malaysian researchers should take a leading role in collaborating on more integrated and multi-disciplinary research so as to enlarge the evidence-base for physical activity promotion.
6. Although awareness of the importance of physical activity is increasing, it often does not translate into actual participation. Policies aimed at increasing access to active transportation and the provision of facilities and opportunities for physical activity at the workplace would contribute toward increasing physical activity levels. Policies providing for tax exemption for the improvement of opportunities for workers to engage in physical activity could help ensure willing acceptance of employers, potentially increasing workplace physical activity.
7. Although there is currently no formal Malaysian professional physical activity network that supports those interested in working in the field, the number of researchers in the area is growing. If there is concerted effort to collaborate on research it could conceivably increase research output.
8. In order to increase Malaysian capacity for physical activity promotion, more co-operation and collaboration amongst stakeholders is needed. This would ensure that ministries, non-governmental organizations, and researchers maximize resources in targeting the widest group of society. Programs need to be inclusive, adopting a life course approach where people of all ages are provided with opportunities to engage in physical activity (35). Programs should also be informed by research and incorporate components of monitoring and evaluation. A more systemic approach toward the promotion of physical activity levels in Malaysia should generate better results. This would ensure their continual improvement and sustainability. In addition, programs should address not only challenges of individual behavior change, but also environmental barriers, if they are to succeed in facilitating a successful transition to a more physically active lifestyle (36).
9. Malaysia should follow the Bangkok Declaration on Physical Activity for Global Health and Sustainable Development. It calls for renewed commitment to decreasing physical inactivity across the lifespan, integration of national action plans on physical activity, training and development of professionals, increased collaboration, and regular monitoring of physical activity, along with the encouragement of collaboration, research and policy evaluation (37).

## Conclusion

This policy brief highlights several opportunities to increase Malaysian capacity for physical activity promotion across all sectors and population groups. Although physical activity surveillance is incorporated in National Health and Morbidity Surveys at intervals of ~5 years, there is need for improvement in the methodology employed. It is very evident that physical activity promotion is emphasized in Malaysia since there are a number of policies relevant to it. These include National Physical Activity Guidelines, and National Strategic Plan for Active Living. Physical activity guidance has even been incorporated within the Malaysian Dietary Guidelines. Beyond this, it may be seen that research on physical activity has increased over the years, comprising both experimental and observational designs, including cross-sectional, longitudinal and case-control studies. With these in place, more commitment and cooperation amongst government and other stakeholders could well be the impetus that drives gradual improvements in physical activity and the health status of the Malaysian population.

## Author Contributions

SK and AR conceived the study. SS and KC collected data. SK, BP, SS, KC, and AR drafted the manuscript.

## Conflict of Interest

The authors declare that the research was conducted in the absence of any commercial or financial relationships that could be construed as a potential conflict of interest.
